# Dermatofibrosarcoma protuberans (DFSP) successfully treated with sorafenib: case report

**DOI:** 10.1186/2045-3329-3-5

**Published:** 2013-04-04

**Authors:** Francois G Kamar, Victor F Kairouz, Alain N Sabri

**Affiliations:** 1 Division of Hematology & Oncology, Clemenceau Medical Center, City Center Building, Suite 3 A, Avenue Nouvelle, P.O. Box 1076, Beirut, Jounieh, Lebanon; 2 Department of Medicine, University at Buffalo, Buffalo, NY, USA; 3 American University of Beirut Medical Center, Beirut, Lebanon

## Abstract

DFSP is a locally invasive, slow-growing tumor of the subcutaneous tissue that rarely metastasizes but recurs frequently after surgical excision. We report herein a case of highly recurrent, locally invasive DFSP that failed both postoperative radiation therapy and complete trial of Imatinib, but was successfully treated with Sorafenib, which showed unprecedented response.

## Background

Dermatofibrosarcoma Protuberans (DFSP) is a locally invasive and slow-growing tumor of the subcutaneous tissue. It rarely metastasizes. It was first described in 1924 as a progressive and recurrent dermatofibroma [1]. DFSP has an annual incidence of only 0.8 cases per million and presents typically at mid-adult life with a slight male predominance [2]. The trunk and proximal extremities are the most frequent locations of the disease, but it can occur at any other site.

DFSPs rarely progress to a high-grade fibrosarcomatous component [3]. One to 4% of patients will develop distant metastasis, typically many years after the development of the initial lesion [4].

According to the NCCN Clinical Practice Guidelines in Oncology, The gold standards treatment is complete surgical excision with appropriate reconstruction [5]. Imatinib is approved for treatment of advanced disease [6-17].

When resection is limited or incomplete, or margins are positive recurrence rate is high [18-20]. In these cases, adjuvant radiation therapy is recommended as well as Imatinib based treatment or enrollment in a clinical trial [5].

We report hereafter a case of highly recurrent, locally invasive DFSP that failed both postoperative radiation therapy and treatment with Imatinib at 400 mg initially that was then increased to 800 mg. The patient received Sorafenib off label and showed an unprecedented response for his disease.

## Case report

A 36 year-old gentleman presented with a left shoulder skin lesion that was initially totally excised. Histopathology reported Dermatofibrosarcoma Protuberans (DFSP). He relapsed sometime after this initial resection and underwent a second surgery followed by several other relapses and re-resections and 2 courses of radiation therapy and ended up having multifocal disease sites on the upper torso, the shoulder and neck. Plastic surgeons exhausted all available skin that could be spared for flaps and reconstruction. After the third relapse and the first round of radiotherapy, he received Imatinib at 400 mg daily with a positive response lasting for about 2 years. Upon progression, he was offered Imatinib 800 mg daily with non negligible side effects and no response.

All pathology readings of repeated excisions were reported as DFSP except for one time, where the presence of cellular pleomorphism and the lack of CD 34 expression raised the possibility of transformation into a fibrosarcoma. Margins following his third resection were always positive.

There was no FISH analysis or RT-PCR or molecular studies ordered to detect the presence of the typical chromosomal translocation t(17;22)(q21;q13) in the tumor of this patient, resulting in the chimeric gene COL1A1-PDGFB.

The patient ended up with left upper extremity amputation at the level of the shoulder and was referred to us.

He presented with massive and bulky soft tissue lesions on his upper torso and in the neck. A contrast enhanced CT scan also revealed large mediastinal masses and post obstructive pneumopathy on the left.

**Figure 1:**
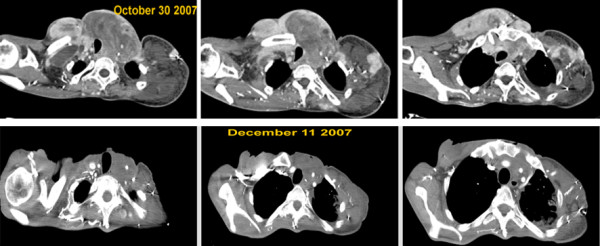
Radiological response to Sorafenib.

**Figure 2:**
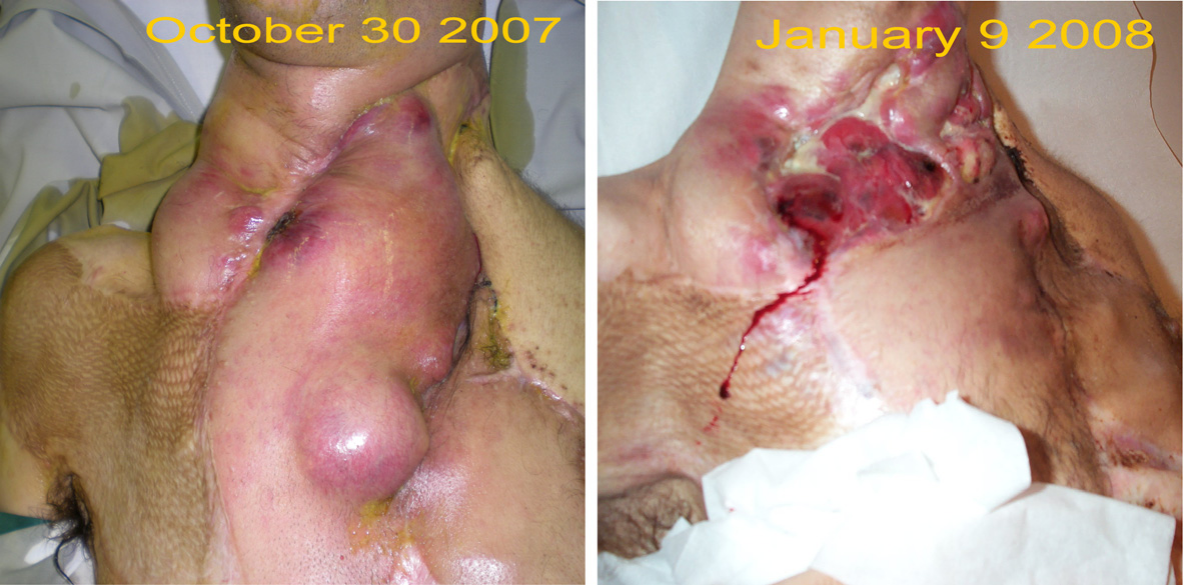
Clinical improvement and dramatic shrinking of the tumor.

He was offered to start on Sorafenib as it was made available for us on compassionate use. A dramatic response was seen after only few days of treatment (Figures 1 and 2). The patient received Sorafenib 800 mg daily for 5 months without recurrence or progression of the tumor. There was no significant side effect reported beside moderate fatigue, and dehiscence of prior surgical wounds. However, he suffered from recurrent pulmonary infections despite decompression of his left main bronchus since attaining a very good partial response, and unfortunately passed away after an episode of septic shock. He remained progression free during the whole treatment time. Therapy was discontinued only one week prior to his death.

## Discussion

DFSP accounts for 2 to 6% of all soft tissue sarcomas [21,22]. It is the most frequent skin sarcoma.

The tumor involves most frequently the trunk (42-72%), the proximal extremities (16%-30%), and the head and neck (10-16%) especially the scalp [23-28].

The initial presentation of DFSP is a skin lesion described as a single, raised, red to bluish, firm cutaneous nodule or plaque with surrounding discoloration [29]. The lesion is painless and indurated, but is extremely infiltrative and has a locally destructive growth that can invade the underlying structure such as fascia, muscles or bones [18-20,30]. The tumor is often covered by a brown-yellow, red-tinged, sclerodermiform or teleangiectatic and atrophic skin [20]. At the initial stages of the disease, history of trauma, burn or surgical scar is often found.

At advanced stages, the tumor can ulcerate, bleed, and become painful, as reported in our case.

Near to 90% of DFSP are considered to be tumors of low-grade malignancy [4]. Histologically, DFSP is identified by a pattern of monomorphous proliferation of cytological bland spindle cells with a visible storiform or whorled (rushmat-like) architecture [4,23,31]. Other characteristic features are low mitotic activity and deep, honeycomb infiltration into subcutaneous adipose tissue [30].

The expression of CD34 is almost a consistent finding and it is extremely useful in differentiation of DFSP from benign fibrous histiocytoma [32], dermatofibroma and other soft tissue tumors [33-35]. The sensitivity of CD34 staining in DFSP ranges from 84 to 100% [30].

Cryptogenic abnormalities are also distinguishable characteristics of DFSP since more than 90% of cases have supernumerary ring chromosomes or a unique translocation involving chromosomes 17 and 22 as in *t*(17;22)(22;q13) [36-41]. This abnormality results in a constitutive stimulation of the platelet-derived growth factor receptor (PDGFR) with subsequent enhancement of the tumor cell growth [6,28]. Furthermore, among all the family of PDGF receptors, this translocation is responsible for the activation of PDGFR-beta [42].

Distant metastasis is rarely observed, with a rate of 4-6%, predominantly to the lungs [20,30].

DFSP can transform, especially in the recurring forms, into fibrosarcomatous DFSP (FS-DFSP), a tumor with higher invasion and malignancy potential [3,4,28,43]. It usually requires a more intensive treatment approach.

The optimal and the mainstay treatment of DFSP, regardless of its location, is wide and deep excision with adequate tumor-free margins. The proper excision with clean margins is directly related to the risk of recurrence of the disease despite adjuvant therapy [18,19,44,45]. The involvement of the deep fascia and muscles requires excision of these structures [18,19].

The most significant prognostic factor in patients with DFSP has proved to be the extent of surgical resection. The success of the initial surgical excision has a major effect on the outcome as well. In fact, if this procedure fails and hence, the tumor recurs, it could lead to an uncontrollable local growth, as seen in our case.

Due to its infiltrative nature, DFSP is characterized by a high recurrence rate varying in the literature from 10-80% [18]. The risk of recurrence was 41% when the excision margin was less than 2 cm and 24% when it was equal to or higher than 2 cm [44].

DFSP is considered as one of the radiosensitive tumors. Earlier studies suggested that additional postoperative RT reduces the risk of local recurrence in patients with questionable or positive surgical margins [46,47]. When complete surgical resection is unachievable, RT is indicated [46,48]. RT can reduce the morbidity or functional impairment associated with extensive resection.

As seen in the present case, locally recurrent DFSP can be devastating. Salvage by further resection increases the risk of functional deficits and metastatic disease.

Since the recent identification of the significance of PDGFR activation in the DFSP pathogenesis, the effectiveness of targeted chemotherapy by inhibition of the PDGFR protein-tyrosine kinase has been evaluated. Imatinib, a small adenosine triphosphate analog is a selective PDGFR tyrosine kinase-inhibitor. It was approved for the treatment of DFSP at a dose of 800 mg daily. Such dose is easily reached orally, with non negligible side effects. Some clinical reports showed Imatinib induced regression in patients with recurrent or metastatic DFSP [6-17].

However, response was not consistent in all cases as described in our patient.

To date, no other targeted therapy for DFSP has been reported.

We report the first case of recurrent and extensive DFSP successfully treated with Sorafenib after failure of postoperative radiation therapy and refractory to treatment with 800 mg of Imatinib.

Sorafenib is a small molecule B-raf and vascular endothelial growth factor (VEGF) receptor inhibitor. It has shown great value in the treatment of angiosarcomas [49] Maki et al., and some other specific sarcoma subtypes (e.g. peripheral-nerve sheath tumors (MPNST) with loss of NF1 and activation of the ras-raf signaling pathway) [50-52].

Since it has been reported to have an important role in the treatment of some sarcomas, we elected Sorafenib as a salvage option for our patient. The response was remarkable. The tumor almost totally regressed and the patient improved clinically. This case suggests that Sorafenib could be an important targeted therapy for DFSP especially in case of recurrence or metastasis.

## Conclusion

Invasive DSFP could be uncontrollable and challenging to cure. We present a case of recurrent and infiltrative DSFP that failed all conventional therapeutic options. Sorafenib was administered as final resort with impressive results.

This is to our knowledge the only reported case of DFSP treated with Sorafenib after failure of Imatinib.

Sorafenib could represent a therapeutic alternative in such cases of DFSP.

## Consent

Written informed consent was obtained from the patient for publication of this report and any accompanying images.

## Abbreviations

DFSP: Dermatofibrosarcoma protuberans; NCCN: National comprehensive cancer network; FISH: Fluorescence in situ hybridization; PDGFR: Platelet-derived growth factor receptor.

## Competing interest

The authors declare that they have no competing interests.

## Authors’ contributions

FGK established the conception, the design, and the interpretation of data. After finalizing the draft, he approved of the version to be published. VFK has made substantial contributions to acquisition, analysis, and interpretation of data. He also was involved in drafting the manuscript and revising it critically for important intellectual content. ANS contributed in data analysis and interpretation, and was involved in revising the critical aspects of the manuscript. All authors read and approved the final manuscript.

## Funding

No external funding, apart from the support of the authors' institution, was available for this study.
